# Experimental study of unconventional modified filling energy absorption and control mechanism in high energy storage rock masses

**DOI:** 10.1038/s41598-022-15954-5

**Published:** 2022-07-12

**Authors:** Xing-ping Lai, Shuai Zhang, Peng-fei Shan, Feng Cui, Yan-bin Yang, Rui Bai

**Affiliations:** 1 College of Energy Science and Engineering, Xi’an University of Science and Technology, X’an, 710054 Shaanxi China; 2 Key Laboratory of Western Mines and Hazard Prevention of China Ministry of Education, Xi’an University of Science and Technology, X’an, 710054 Shaanxi China

**Keywords:** Civil engineering, Seismology

## Abstract

Aiming at the problems that it is difficult to predict rock burst accurately in engineering practice and the implementation parameters of rock burst prevention measures depend on some empirical formulas, in order to study the advantages and disadvantages of different in-situ modification mechanisms deeply, determine the applicable conditions of unusual in-situ modification measures, and provide a theoretical basis for forming adaptive in-situ modification control schemes. Two kinds of modified control methods using the same foundation involve engineering scale and indoor scale. With the help of scale transformation, the whole failure process analysis test of bearing rock samples was carried out. The results show that various modification measures can effectively control the properties, and realize "hard-rock softening or soft-rock hardening" by changing the physical and mechanical parameters of the target rock sample. Compared with the control group, the automatic parameters of rocks deteriorated significantly under different modification measures. The evolution law of carrying energy is similar. However, there are obvious diversity between various modification measures in plastic stage and post-peaking phase stage, which provides favorable conditions for rock burst prevention. Based on this, an adaptive modification control system was constructed. At the same time, filling materials is considered to increase the energy of post-peaking phase (non newtonian fluid: energy-absorbing materials), and further slow down the intensity of released energy within post-peaking phase stage. Because rock burst is characterized by rapid release of energy, non newtonian fluid has a good absorption effect on high-speed impact force. Therefore, in the design test, the effect of non newtonian fluid is realized by applying a high loading rate, and the evaluation of energy absorption effect of bearing rock samples filled with non newtonian fluid in borehole is considered.

## Introduction

The introduction of the dual carbon policy has stepped up the pace of energy restructuring in China. However, due to the characteristics of China's resource endowment, it determines that China will still use coal as the primary energy source for quite some time. To respond positively to the dual carbon policy, China will develop specific measures, which will inevitably put the efficient and clean use of coal to a severe test^1^. Therefore, The exploitation of coal resources is particularly consequential. As coal resource mining in China goes deeper, frequent dynamic disasters have severely restricted the safe mining and stable supply of coal, among which the dynamic disasters represented by rock burst are the most significant^2–4^. Scholars at home and abroad have carried out extensive research on this global problem, mainly focusing on the following aspects: Firstly, some scholars, while improving the existing mechanism for the occurrence of rock burst, still dig deeper into new theories to try to provide a reliable description of the formation mechanism of rock burst and explain the whole process of rock burst conception^5–9^. Secondly, considering that the formation of rock burst is inevitably associated with high-stress concentration, some scholars have studied the stability of the surrounding rock under various pressure relief measures and used a variety of standard monitoring equipment to assess the reasonable effectiveness of different pressure relief measures^10–14^. Finally, as the destruction of coal rock samples is a highly non-linear process, a single evaluation index is often unable to invert its entire destruction process effectively. The research work inevitably requires the use of methods such as big data, cloud mining, and artificial intelligence, specifically with the aid of multiple monitoring devices (acoustic emission, thermal infrared, electromagnetic radiation, etc.). By conducting indoor laboratory-scale tests to study the damage pattern of single and combined bodies of load-bearing coal rocks under various loading paths, and identifying the reliable inception signals of damage precursors, mainly focusing on the pick-up of multi-physical quantities, to provide a basis for further research on inception signals^15–20^. In turn, the mature indoor theories can be extended to practical engineering scales, and also provide strong evidence for the prevention of rock burst accidents in the field. However, due to the many factors involved in rock burst and the complex occurrence mechanism, especially the prediction and control of dynamic hazards under the mining of “long distance–large scale–high strength” is particularly difficult, which brings unprecedented challenges to deep coal resources mining and underground engineering maintenance. These will be the key research contents in the next step.

At present, the idea of rock burst research with energy conduction as the mainline is widely accepted by many scholars. The essence of rock burst formation is the problem of instability caused by exceeding the threshold value acting on the energy storage during the equilibrium of stress redistribution under the influence of mining disturbance^21^. Considering that there are more disaster-causing mechanisms of rock burst, this paper only discusses strain-mode rock burst. From the above analysis, it can be seen that the accumulation and release of energy in the coal rock masses are reasonable or not, which directly determines whether the rock burst can be accurately warned and effectively controlled. Referring to the concept of in-situ modification put forward by Academician Zhao Yangsheng, this paper introduces it into the study of rock burst control mechanism. At present, in terms of in-situ modification methods and control mechanisms, the main techniques of rock burst regulation and control are as follows. Water injection reduces the strength of the coal rock body and acts as a pressure relief. Blasting consumes the total energy after the initiation of rock burst and reduces the rate of energy release, thus reducing the degree of rock burst hazard. Large-diameter hollow holes lessen the accumulation of elastic energy in the rock masses above and around the roadway, paring the regional impact hazard. Adjustment of the mining layout can increase energy dissipation. The use of energy absorption devices can also reduce the probability of rock burst disasters. Filling control the deformation of the roof plate and the accumulation and release of energy, etc. These methods and explanations of the mechanisms of rock burst occurrence have been influential in the corresponding dynamic disasters control engineering practice. We could see that these studies analyzed the adjustment of stress concentrations by control techniques, mostly without analyzing the coupling between control methods and energy regulation. In contrast, numerous analytical methods lacked a solid theoretical basis, which led to practical measures in the coal often relying more on experience to determine specific regulation parameters^22,23^.

Better explaining the corresponding energy regulation mechanism in rock burst, this paper takes the commonly used measures (water injection softening and large diameter pressure-relief borehole) as an example, and designs two parallel test ideas for rock samples with different water content and different pore size parameters. With the help of scale transformation, this paper analyses the mechanical properties and parameters during the unbearing loading of rock samples in different conventional modification states with indoor uniaxial compression tests, and investigates the evolution of energy characteristic parameters during the unbarring loading of rock samples in other ancestral modification states. Further, this paper presents a comprehensive analysis of the energy regulation mechanism of traditional modifications and the advantages, disadvantages, and applicability of each regulation measure to provide a theoretical basis for the reasonable determination of an adaptive integrated regulation system. Finally, By introducing non newtonian fluid energy-absorbing materials to fill the large diameter unloading borehole, this paper effectively absorbs the elastic energy released from instability. The research results are of great theoretical value and engineering significance for understanding the energy regulation mechanisms of rock masses in different modification states and improving the theory of rock energy and the theory of artificially induced regulation of dynamic hazards.

## Test scheme

### Engineering background and problem introduction

The Kuangou coal mine of Xinjiang Energy Tiandian Mining Industry is a typical rock burst mine. Given the complexity of the mechanism of the occurrence of rock burst, combined with the actual production, through on-site monitoring, indoor tests, and numerical simulation, it was further clarified that the overlying hard roof is the dominant factor inducing the rock burst in the Kuangou coal mine. According to the national standard, both the coal and the roof are judged to have a substantial impact propensity. Due to the unique properties of the complex top, the hard roof accumulates more elastic energy after the redistribution of stresses under the effect of mining disturbance. Once the instability causes the immediate release of elastic energy, it will induce rock burst. Therefore, from the perspective of engineering practice, it is necessary to focus on the hardtop slab and strengthened the pressure relief management of the full slab to avoid high-stress concentration^24^.

### Sampling position and rock sample preparation

**Figure 1:**
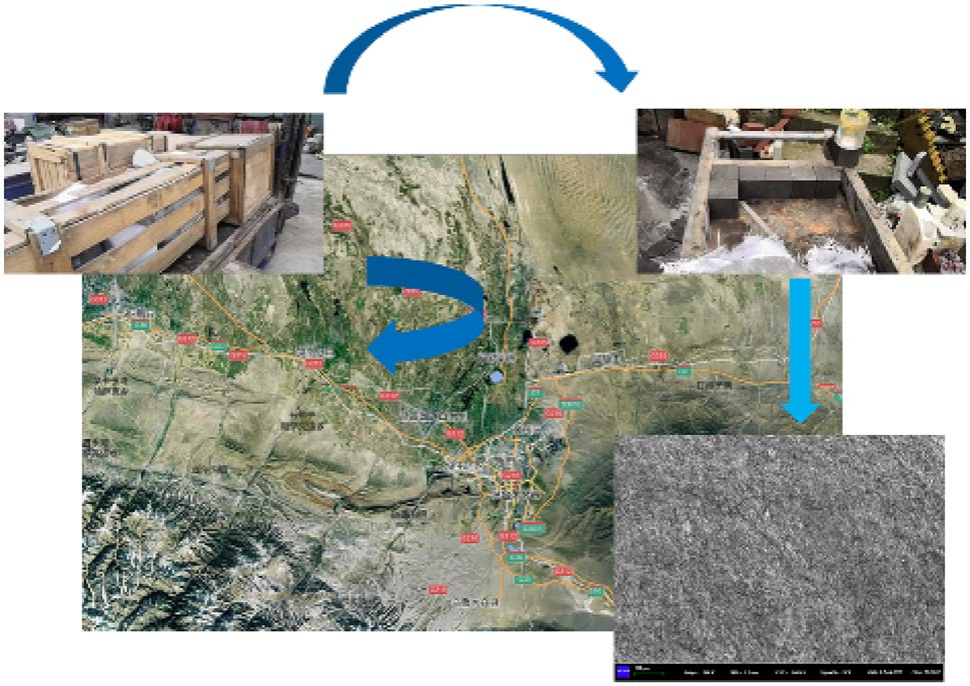
Sampling position and scanning electron microscope results.

Based on the available analysis, the rock sampling locations used in this test were taken from the W1123 working face of Kuangou coal mine of Xinjiang Energy, and the sampling locations are shown in Fig. 1. According to the relevant requirements, the roof slab was sampled at the back of the working face support in the height direction perpendicular to the seam joints. Hexahedral sandstone samples of roughly 30 cm × 30 cm × 30 cm in size were obtained and transported to the laboratory for subsequent processing. By the ISRM test standard, the samples were processed into 70 mm × 70 mm × 70 mm square samples with a flat surface and no obvious cracks, and the cut clayey siltstone was polished with fine sandpaper to ensure that tolerances were allowed.

### Rock sample microstructure and test system

The microstructure of the rock sample was observed with the aid of a scanning electron microscope (SEM). The specific SEM image of the rock sample is shown in Fig. 1. The results show that the model is dense, with the grains forming bridging growths in the pores, forming wispy, hair-like networks. When the quartz grains are magnified, a long, regular crystal structure is evident, with a large amount of slab-like kaolinite and filamentous illite distributed on the grain surface. Based on multiple sets of scanning electron microscopy without magnification, it was judged to be a complex rock formation.

**Figure 2:**
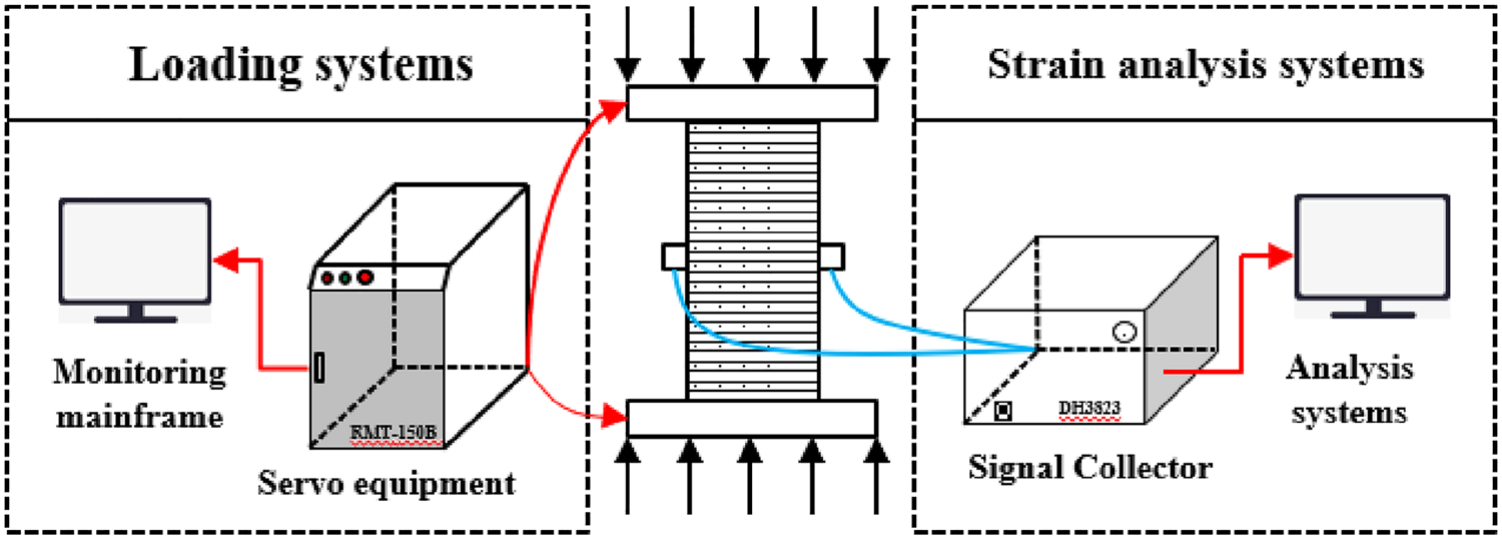
Rock mechanics test system.

The test was carried out with a self-built integrated test system, consisting of a loading control system, a strain analysis system, and asynchronous video acquisition system. The loading control system was implemented by the RMT-150B rock mechanics testing machine. The strain analysis system is implemented by the DH3823 distributed signal test and analysis system. The video synchronization system is implemented by a complete process video capture device. To ensure the reliability of the test data, the rock sample is loaded using controlled stress at a constant rate of 0.2 kN/s. The test monitoring software automatically collects and processes the test data in time, displaying the stress-displacement curve, stress-time curve, displacement-Time curve, etc. The test system diagram is shown in Fig. 2.

## Mechanical properties of rock samples carried by different modifications and controls

### Homologous modification and modulation scale transformation of engineering measures and indoor solutions

To simulate as comprehensively as possible several commonly used in-situ modification and restore them to the natural engineering environment in an infinitely close manner, the mechanisms of various in-situ modification are discussed and attempted to be clarified. The advantages and disadvantages of various in-situ modification measures are investigated in-depth. The conditions of application of additional in-situ modification measures are analyzed to provide a theoretical basis for the formation of an adaptive in-situ modification and control scheme. Based on the above analysis, the contribution of the influencing factors to the study's findings is also identified with the help of a variety of well-developed research tools from indoor experiments. In this paper, the influencing factors that contribute to a lesser extent are approximately reduced and transformed to study the variability of damage evolution patterns of different measures on the scale of indoor experiments. The findings provide data support to reveal different in-situ modification modulation mechanisms in an engineering scale context.

In conjunction with the actual production situation at Kuan Gou, the mine has developed a series of anti-surge and anti-danger measures based on real-time analysis of the impact precursors of the mining impact. These measures include blasting (indoor studies are impossible due to lack of test conditions), hydraulic fracturing, and large diameter pressure releasing borehole. Therefore, this paper is based on the above measures, transformed into indoor experiment's scale study of the differences in the mechanisms of different modification measures on rock samples. The details are as follows.

1. Engineering scale water injection softening regulation (corresponding to indoor test scale change of relative water content parameter).

**Figure 3:**
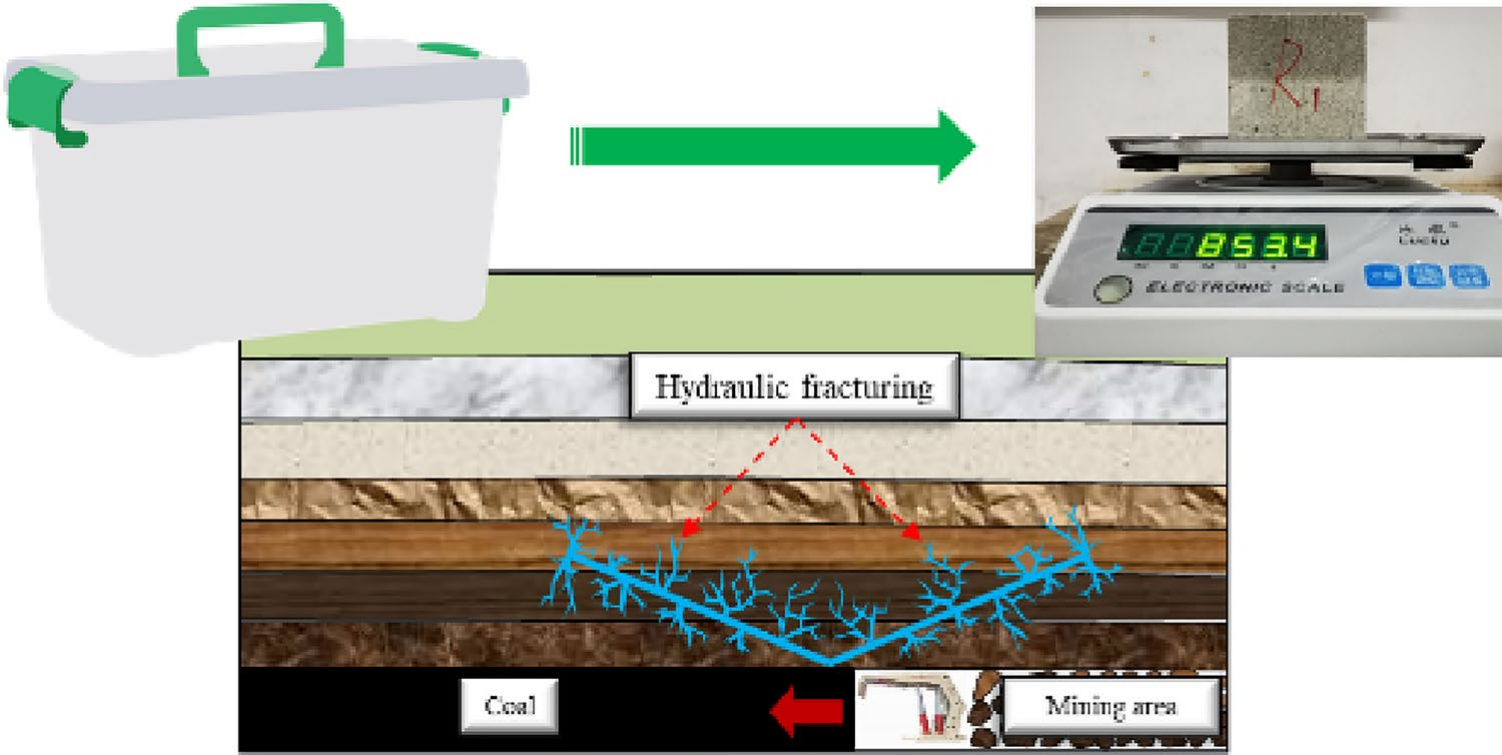
Control measures of different water-bearing states.

In this paper, to simulate the water injection softening engineering background, as shown in Fig. 3, in an indoor laboratory environment, the coal sample specimens were artificially set up by immersion in different water content states. To avoid interference from other factors, the coal rock samples taken and shaped were air-dried for 24 h and defined as the natural state, which was approximated as the same water content in the same condition. In addition, drying and soaking were used to obtain coal samples less than and more significant than the natural state according to the water absorption and water loss rules. The specimen numbers and the different water content states are shown in Table 1.

(2) Engineering scale extensive diameter pressure relief borehole regulation (corresponding to indoor test scale change of relative borehole parameters).

**Table 1:** Sample number and moisture content in each state.

State of the specimens	The sample name	Specimen number	Average moisture content
Dry state	Rock sample	R_0-1, 2, 3_	0.00%
Nature state		R_1-1, 2, 3_	2.41%
Saturated state		R_2-1, 2, 3_	3.83%

**Figure 4:**
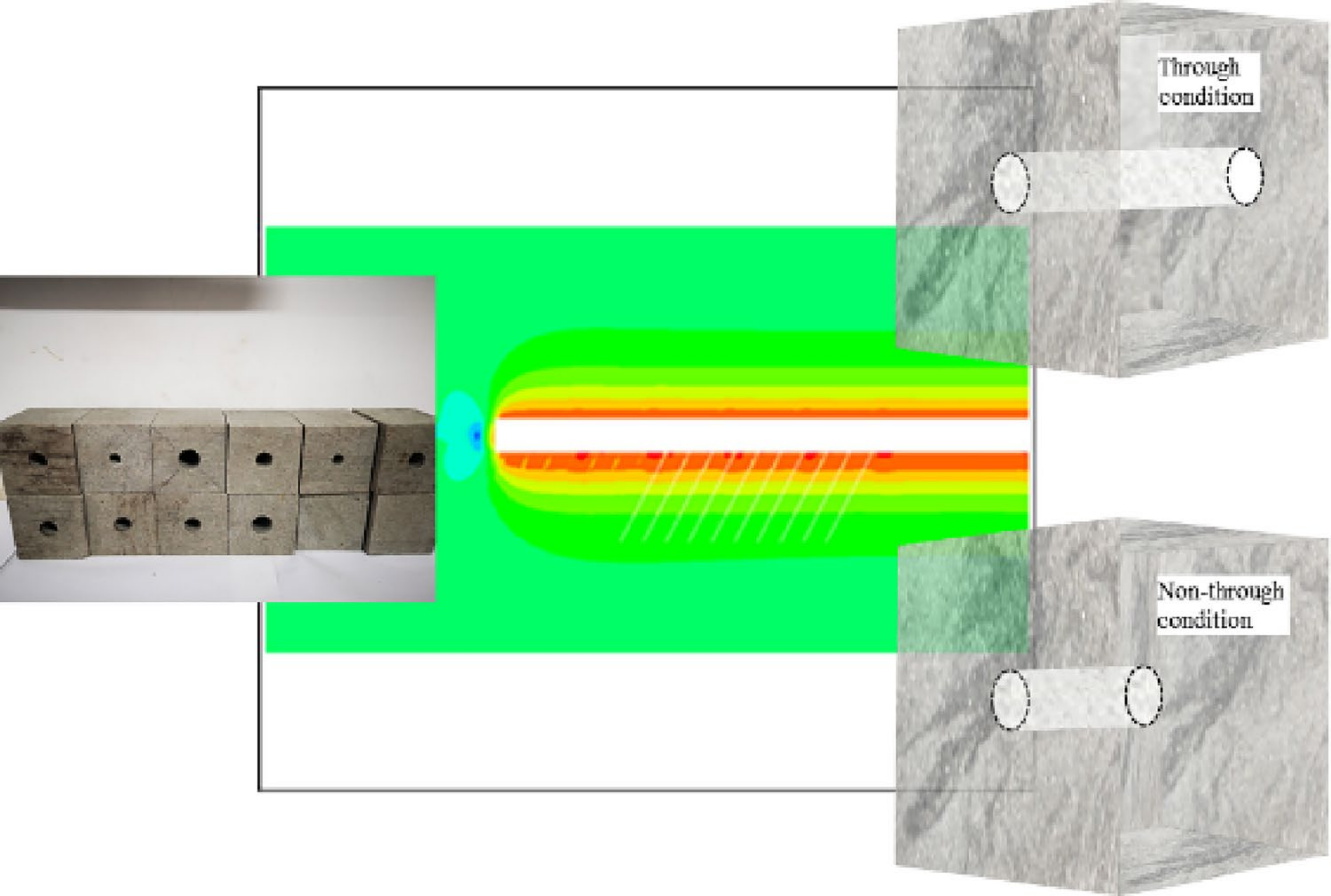
Control measures of different drilling states.

To simulate the engineering context of large-diameter pressure relief boreholes, this paper carries out experiments, as shown in Fig. 4. Considering the limitations of field boreholes, the problem of contact between the borehole end and the solid rock that exists, the study is therefore carried out in two distinct aspects: borehole through and borehole non-through states. Furthermore, to compare the variability of the unloading effect of different borehole diameters of rock, rock samples of varying borehole sizes and different degrees of penetration were artificially set up through the borehole in an indoor laboratory environment.

In the process of bearing, the borehole rock has obvious boundary effect. To reduce the influence of the boundary effect on the test results, the parameters were determined according to the theories and some empirical findings of the relevant scholars. The general boundary size is greater than five times the radius of the hole, so this test set two hole size samples as different hole sizes through state specimens, that is, respectively, the radius of the hole formed to r_1_ = 5 mm, r_2_ = 8 mm. The specimen number and different drilling state are shown in Table 2.

**Table 2:** Sample number and drilling parameters of each state.

State of the specimens	The sample name	Specimen number	Drilling parameters
Small aperture penetration state	Rock sample	R_3-1, 2, 3_	r_1_ = 5 mm
Large diameter through state		R_4-1, 2, 3_	r_2_ = 8 mm
Small aperture non-penetration state		R_5-1, 2, 3_	r_1_ = 5 mm d = 60 mm
Large aperture non-penetration state		R_6-1, 2, 3_	r_2_ = 8 mm d = 60 mm

### Stress–strain curves for load-bearing specimens with different modifications and control measures

In this test, uniaxial compression tests were carried out on rock samples under different modification and control measures, and the mechanical parameters were processed and counted as shown in Table 3.

**Table 3:** Average mechanical parameters of rock samples under different modification states.

Corresponding working condition	State of the specimens	Processing state	Specimen number	Average peak intensity/MPa	Average peak strain	Average modulus of elasticity/GPa
Blank test	Nature state	Blank untreated	R_0-1, 2, 3_	28.20	0.02127	2.42
Water softening	Dry state	Drying state	R_1-1, 2, 3_	44.56	0.02564	3.56
	Saturated state	Saturating state	R_2-1, 2, 3_	22.07	0.02120	1.79
pressure relief borehole	penetration state	Small aperture penetration state	R_3-1, 2, 3_	25.06	0.02597	0.86
		Large aperture penetration state	R_4-1, 2, 3_	16.57	0.01969	1.27
	non-penetration state	Small aperture non-penetration state	R_5-1, 2, 3_	17.24	0.02621	1.58
		Large aperture non-penetration state	R_6-1, 2, 3_	22.49	0.02011	1.62

To compare the differences of various modification measures, this paper, firstly, analyses the stress–strain curve of typical bearing rock samples in their natural state. Secondly, based on the engineering mentioned above, it is proposed to compare the stress–strain curve of standard bearing rock samples before and after the implementation of the following three different modification measures for specific comparative analysis.

1. Water injection softening control

**Figure 5:**
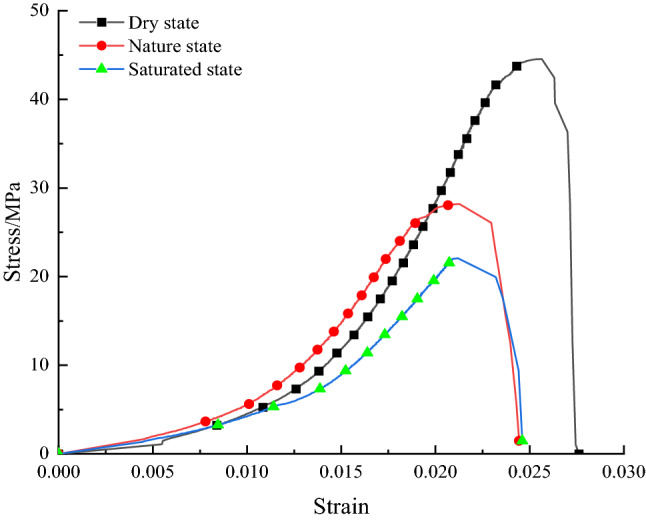
Stress–strain curves of typical rock samples in different water-bearing states.

Figure 5 shows the stress–strain curves of the rock samples under different water injection softening control (changing the water content). The test results show that the uniaxial compression stress–strain curves of the rock samples under separate water injection and softening control states have good similarity. In contrast, the post-peak stress–strain curves differ, and the post-peak strain range shows a positive correlation with the water content.

For the water injection softened modulated rock samples, the average peak strengths were 44.56 MPa, 28.20 MPa, and 22.07 MPa for 0, 2.41%, and 3.83% water content, respectively; the average peak strains were 0.02564, 0.02127, and 0.02120, respectively; and the average moduli of elasticity were 3.56 GPa, 2.42 GPa, and 1.79 GPa, respectively. Compared to the thoroughly dried rock samples, the average peak strength decreased by 36.7% and 50.5%. The average peak strain decreased by 17.0% and 17.3%, and the average modulus of elasticity decreased by 32.0% and 49.7%, respectively.

Comparing the test results, it can be seen that as the water content increases, the peak strength decreases, the peak strain decreases, and the modulus of elasticity decreases, but the reduction significantly reduces with increasing water content.

As the water content increases, the uniaxial compression test stress–strain curve tends to shift to the left during the whole process. The reason for this is that the water molecules in the fissures weaken the cohesion between the fissured particles, allowing the rock sample to be softened. This factor reduces the peak strength and modulus of elasticity of the rock sample, which is macroscopically manifested by a reduction in the overall mechanical properties. However, the peak strain also shows a negative correlation due to the unique nature of the complex rock formations, which have maintained their brittle damaged properties. As the water content increases, the range of post-peak strain intervals increases further, showing some flexibility. In addition, the decrease in each mechanical parameter decreases significantly with increasing water content.

(2) Large diameter borehole decompression control

**Figure 6:**
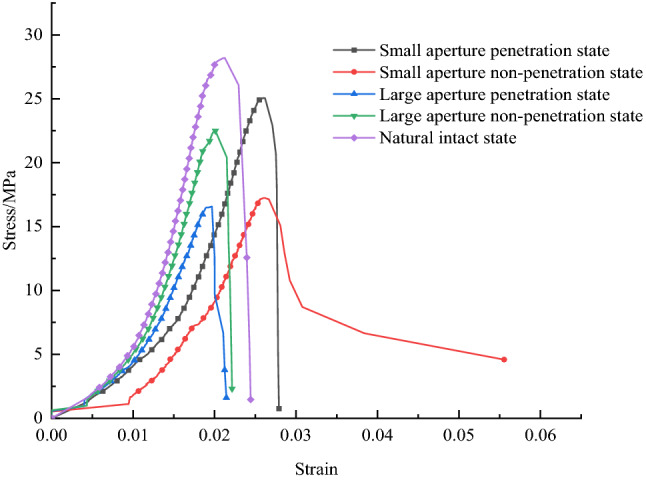
Stress–strain curves of typical rock samples with different drilling parameters.

Figure 6 shows the stress–strain curves in different borehole decompression control (changing borehole size) states. The test results show that the stress–strain curves in uniaxial compression tests in other borehole decompression control states have good similarities. Still, the mechanical parameters of characteristic points show differences as the borehole size changes.

In the case of the borehole decompression control samples (where the natural state samples from the water injection softening control were used as the intact state samples for blank comparison analysis), the average peak strengths were 28.20 MPa, 25.06 MPa, and 16.57 MPa for hole diameters of 0 mm, 10 mm and 16 mm, separately. The average peak strains were 0.02127, 0.02597 and 0.0197, separately. Compared to the natural intact rock samples, the average peak strength decreased by 11.1% and 41.2%, the average peak strain increased by 22.1% and decreased by 7.4%. The average modulus of elasticity decreased by 64.6%, and the average modulus of elasticity decreased by 64.6% and 47.5% respectively.

The test results show that the peak strength of the rock samples continues to decrease as the hole size increases, and the magnitude of the decrease increases significantly with the increase in hole size.

Compared to the natural intact rock samples, the peak strength of the borehole decompression-controlled rock samples is reduced. By changing the borehole diameter parameters, the mechanical parameters change significantly. This is reflected in the fact that small borehole diameters correspond to an increase in breaking strain, while large borehole diameters correspond to a decrease in breaking strain. The reason for this is that the stresses are adjusted during the bearing process for samples containing holes. The small aperture samples are more fully adjusted to the stresses and form greater strains during the stress adjustment process, while the large aperture samples are not adjusted to the greater stresses before the overall damage occurs, corresponding to a decrease in the damage strain. The presence of holes in the samples led to increased initial damage and reduced strong brittle characteristics, but due to the artificially constructed damage surface, all damage cracks intersected in the area of the holes, thus providing some directional relief.

## Energy evolution patterns of rock samples carried by different modifications and control measures

Based on the broad consensus that energy release induces the formation of rock burst, the energy principle is generally applied to the analysis of the energy evolution pattern of bearing rocks^25^.

### Energy distribution characteristics of rock samples under different modification measures

In this experiment, uniaxial compression tests were carried out on rock samples in other modified states to analyze their energy evolution patterns. The energy distribution of the rock samples in the intact natural state is, firstly, plotted. In addition, to compare the differences between the various modification measures, the analysis is carried out in the following three various modification measures.

1. Typical natural intact state

**Figure 7:**
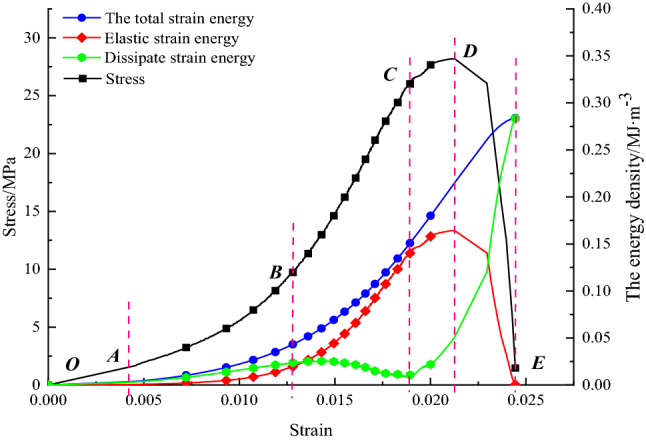
Energy density curve of natural rock samples.

Figure 7 shows the relationship between stress, energy, and strain in the natural shape of the rock sample under uniaxial conditions. The loading process of the loaded rock sample goes through five stages: initial compaction stage (OA), elastic deformation stage (AB), plastic deformation stage (BC), yielding damage stage (CD), and late damage stage (DE).

(2) Water injection and softening modulation

**Figure 8:**
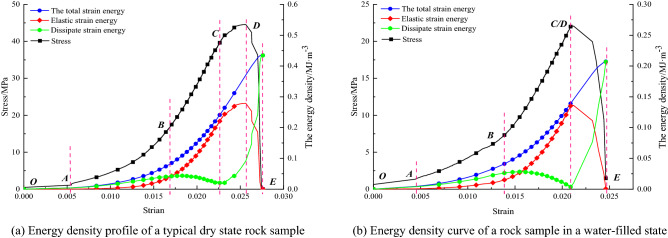
Relationship between stress, energy, and strain of rock samples under uniaxial conditions of different water-bearing states.

Figure 8 shows the relationship between stress, energy, and strain in the dry and water-filled states of the rock sample under uniaxial conditions.

The energy evolution curves show that the energy components of the rock samples with different water contents show corresponding changes at various stages of the stress–strain curve. This includes the initial compaction stage (OA), the elastic deformation stage (AB), the plastic deformation stage (BC), the yielding failure stage (CD) and the late failure stage (DE). In the dry state, the energy dissipation curve is fundamentally different from that of the natural and waterlogged state. In the dry state, the curve of energy dissipation density shows a decreasing inflection point and then slowly increases. In the wild, soggy state, the dissipative energy density curve shows a significant downward trend followed by a sudden increase.

(3) Pressure relief control in large diameter boreholes

**Figure 9:**
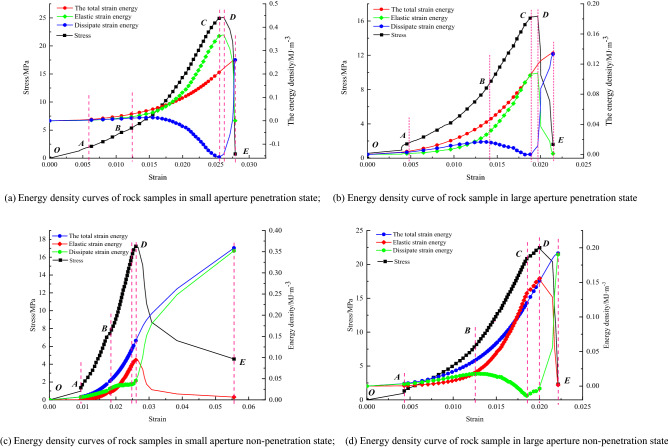
Relationship between stress, energy, and strain of rock samples in different drilling states under uniaxial condition.

Figure 9 shows the relationship between stress, energy, and strain for slight borehole penetration, extensive borehole penetration, small borehole non-penetration, and large borehole non-penetration under uniaxial conditions. The energy evolution curves can be summarized to show that the energy components of the different apertures show corresponding changes at various stages of the stress–strain curve, compression-density, elasticity, yielding, and damage stages. In the natural and large pore sizes, the elastic energy density curve is lower than the total strain energy curve.

### Analysis of the energy consumption characteristics of rock samples under different modification measures

1. Water injection softening regulation

**Figure 10:**
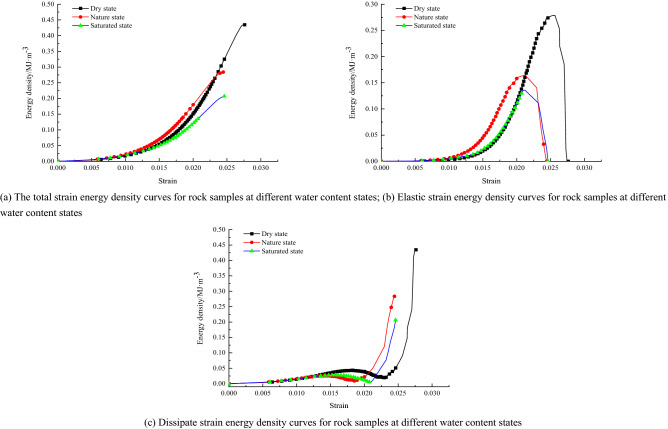
relationship curves of total strain energy, elastic strain energy, and dissipative strain energy with the strain of rock under different water-bearing conditions.

The curves of total strain energy, elastic strain energy, and dissipative strain energy versus strain for rock samples with different water content states are given in Fig. 10, respectively. It can be found through the curves than with the increase of water content, the total energy absorbed by the rock sample slows down with the growth rate of strain. In contrast, the peak point of elastic strain energy of the rock sample keeps decreasing with the increase of water content. The softening effect of water on the rock leads to a reduction in the cohesion of the cohesive particles. The water disrupts the internal micro mechanical structure, allowing the rock to reach a lower energy state.

In the dry state, during the compression and elastic stages, the energy absorbed by the rock sample is mainly stored in the form of elastic energy. Only a very small part of it is used for energy dissipation, at which time the elastic energy curve is comparable to the total energy curve, while the dissipation energy curve is low and smooth. After entering the yielding stage, the elastic energy curve increases at a slower rate (a decreasing inflection point), in contrast to the dissipation energy curve, which increases at a quicker rate, indicating that the total energy used for plastic strain. In the damage phase, the elastic energy decreases rapidly with the brittle fall of the stress curve, the elastic energy stored in the rock is released instantaneously, and the dissipated energy used for further development of rock rupture and shear deformation in the slip plane continues to increase, and almost all the total energy absorbed in this period is converted into dissipated energy consumption. In the case of water content (in the nature and saturated state), the change in the energy curve of the rock sample during the compression and elastic stages is consistent with the dry state. However, in the yielding phase, the elastic energy curve increases at a faster rate. In contrast to the dissipative energy curve which decreases abruptly (corresponding to the calm storage period), indicating that more and more elastic strain energy is accumulated, the proportion of elastic strain energy increases rapidly and the ratio of dissipative strain energy. In the damage phase, the elastic energy decreases quickly with the brittle fall of the stress curve, the elastic energy stored in the rock is released instantaneously. The dissipative energy used for the further development of rock rupture and shear deformation in the slip plane continues to increase, with almost all the total energy absorbed during this period being converted into dissipative energy consumption.

Through analysis, this situation may occur due to the presence of water, after the compression and elastic stages, the pore water coupled with the rock matrix to form a more homogeneous elastomer. The water softened the rock matrix at the same time, but also a large amount of elastic strain energy absorption, further resulting in the water-bearing rock samples loaded yield stage elastic strain energy share increased, corresponding to the accumulation period (acoustic emission calm period), when its pore water pressure increased to the fracture. When the pore water pressure has increased to the extent that it extends and penetrates the fracture, the specimen immediately enters the damage phase.

(2) Borehole decompression control

**Figure 11:**
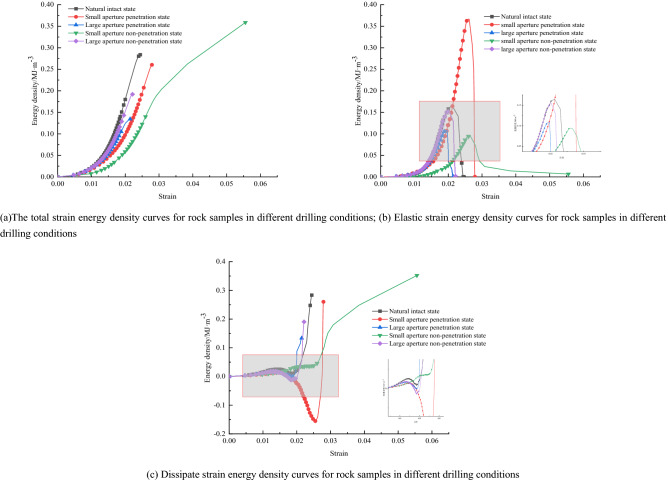
Relationship curves of total strain energy, elastic strain energy, and dissipative strain energy with the strain of rock under different water-bearing conditions.

Figure 11 shows the relationship between total strain energy, elastic strain energy, and dissipative strain energy and strain for the rock samples in different hole states. During uniaxial compression, the rock sample in the unfilled shape of the borehole, due to its hole defects, is prone to stress aggregation around the hole, which leads to rapid crack initiation and expansion and other chain damage reactions, and the sudden and immediate release of the elastic strain energy stored before the original peak.

The curve shows that the elastic energy density curve of the rock sample in different drilling conditions is distinguishable. With the size of the borehole changing, the total energy absorbed by the rock sample changes significantly with the growth rate of strain, and the peak point of the total strain energy of the rock sample also changes. The total strain energy is ranked as follows: small aperture non-penetration state > natural integrity state > small aperture penetration state > large aperture non-penetration state > large aperture penetration state. The elastic strain energy of a rock sample can be divided into an energy storage phase and an energy release phase during the whole process of uniaxial compression. The differences in the energy storage phase of the elastic strain energy of the rock sample are complex. They will be analyzed in conjunction with the acoustic emission properties, and therefore, will not be discussed in this paper. The release phase of the elastic strain energy of the rock sample is significantly different, and the release process of the elastic strain energy is ranked as follows: small aperture penetration state > natural intact state > large aperture penetration state > large aperture non-penetration state > small aperture non-penetration state.

From the above multiple sets of plots, it can be seen that typical rock samples in the nature state, there is a significant initial compression and density stage (OA). Showing a downward trend, and the energy density of the three in this stage is characterized by a non-linear increasing relationship with strain, because the existence of natural random microfractures leads to a greater growth rate of dissipative strain energy than elastic strain energy at the beginning of loading. Which is because the initial deformation stage, microfracture closure and friction in the rock body consume this is because most of the energy is consumed by micro crack closure and dissent in the initial deformation phase; thereafter, the elastic deformation phase (AB) is entered, where the stress–strain curve is basically linear, so that the elastic strain energy starts to become dominant during this phase, and both the total and elastic strain energy started to be characterized by a more constant rate of an increase with increasing strain, and obviously the rate of growth of the dissipated strain energy gradually slows down. The total strain energy input at this stage, contributes a significant proportion to the elastic strain energy of the rock sample, so that the dissipated strain energy is stable within a specific range. During uniaxial compression, the total strain energy and the elastic strain energy, both continue to increase as the strain increases. Still the, elastic strain energy rises rapidly and reaches an extreme value at the yield strength point. The dissipative strain energy starts to fall quickly at this stage. The dissipative strain energy curve shows a clear "concave" trend, which corresponds well to the "storage period" before the failure of brittle rock masses. This is because, as the deformation progresses, all the micro cracks present in the friable rock sample are closed and have good integrity, and continue to exert their energy storage properties. In the yield damage stage (CD), after the yield strength point and up to the peak strength point, the degree of damage to the rock sample increases rapidly. The dissipated strain energy rises quickly, and the stored elastic strain energy decreases slowly with the stress drop. The number of micro cracks increases as new micro cracks are progressively created within this stage. In the late damage stage (DE), after the peak strength point, the releasable elastic strain energy stored before the peak is rapidly released, the dissipative strain energy then increases quickly, and the elastic strain energy decreases quickly. At this stage, the post-peak releasable elastic energy is assisted by generating macroscopic cracks, accompanied by kinetic energy, etc.

## Analysis of conventional modification and control mechanisms and determination of adaptive control systems

### Analysis of the difference in strain ratios for different modification and regulation measures

**Figure 12:**
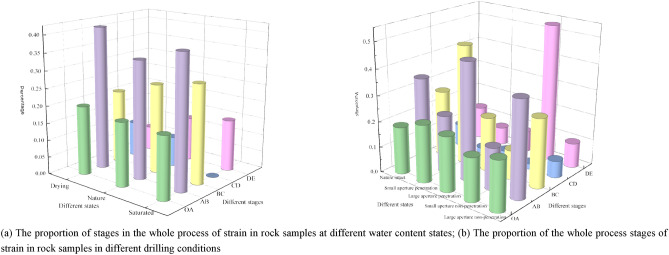
Comparison of proportion contribution of rock sample strain in the whole process under different modification states.

From the above analysis, it can be seen that the elastic energy consumption ratio under different modification and control measures can predict the appearance of the yield point C, and additional modification and control actions have an impact on the proportion of different stages. In contrast, whether the rock burst can be accurately and effectively predicted, in addition to the yield point C, also depends on the proportion of the yield damage stage (CD), that is, the longer the yield damage stage (CD), the more influential the rock burst prediction signal. Therefore, the strain ratios of each step of the damage process of the bearing rock sample under different modification and control measures were calculated, focusing on the percentage of the yielding damage stage (CD) and plotting the percentage contribution as shown in Fig. 12.

As it can be seen from Table 4, as the water content increases, the proportion of yield damaged stage (CD) becomes lower, because water molecules reduce the peak strength and peak strain of the rock sample by softening the friction between the rock matrix, which is further characterized as reducing the brittleness of the rock sample. The reduction increases with increasing water content, while reducing the proportion of yield damage stage, which poses some risk to accurate field engineering predictions.

**Table 4:** Strain ratio of different stages in the whole failure process of bearing rock samples under other water-bearing conditions.

Specimen condition	Initial pressure density stage (OA)	Elastic deformation stage (AB)	Plastic deformation stage (BC)	Yield damage stage (CD)	Destruction of late stage (DE)
Dry state	20.0%	41.3%	21.4%	10.2%	7.1%
Nature state	18.5%	34.1%	25.9%	8.7%	12.8%
Saturated state	18.0%	38.4%	28.6%	0.0%	15.0%

As it can be seen from Table 5, compared to the natural state, as the hole diameter increases, the proportion of yield damaged stage (CD) first decreases and then increases. The reason for this is that in the preload bearing stage of small aperture rock samples, the self-stability of the rock sample is achieved through stress adjustment, during which cracks continue to sprout, characterized by the production of more considerable axial deformation, which contrasts with the increase in peak strain in this state, reaching the yield point C, the subsequent rock mass rapidly destabilization. In contrast, in the preload phase of large-bore rock samples, the initial damage is extensive. Stress adjustment is inadequate, crack sprouting is not developed. There is still a tiny yield breakdown phase (CD) followed by rapid destabilization of the rock mass when the yield point C is reached. Therefore, significant aperture unloading is helpful for the accurate prediction of field projects.

**Table 5:** Strain ratio of different stages in the whole failure process of bearing rock samples under other hole states.

Specimen condition	Initial pressure density stage (OA)	Elastic deformation stage (AB)	Plastic deformation stage (BC)	Yield damage stage (CD)	Destruction of late stage (DE)
Nature intact	18.5%	34.1%	25.9%	8.7%	12.8%
Small aperture penetration	22.5%	22.3%	46.3%	1.9%	7.0%
Large aperture penetration	21.3%	45.3%	21.1%	4.0%	8.3%
Small aperture non-penetration	16.9%	16.4%	11.4%	2.1%	53.2%
Large aperture non-penetration	19.5%	37.3%	26.9%	6.6%	9.7%

### Energy regulation mechanism for "Release first, then weaked" water injection softening measures

The water molecules in the fractures weaken the cohesion between the fracture particles, allowing the rock sample to be softened. The water reduces the peak strength and modulus of elasticity of the rock sample, which is macroscopically manifested by a reduction in the overall mechanical properties. The peak strain also shows a negative correlation due to the specific nature of hard-rock formations, which have maintained their brittle damage properties. However, the reduction in the brittleness of the rock sample is accompanied by a reduction in the proportion of yielding damage stages, which poses some risk to accurate predictions for field engineering. It is suitable for overall modulation on a large scale in the field.

### Energy regulation mechanism for "Release first, then guided" borehole pressure relief measures

In terms of the whole condition, compared to an undrilled intact rock sample, drilling inevitably leads to a significant reduction in the peak strength of the whole rock sample compared to an undrilled entire rock sample. In contrast, the peak strain is similarly reduced, so purely in terms of the pre-peak phase of the stress–strain curve. The elastic energy stored in the whole rock sample before the peak is reduced, and the method matches the idea of pressure relief for managing rock burst. However, because of the artificially constructed soft surface, it inevitably reduces the material's own energy storage threshold, and because of the brittle nature of the material, leads to a sharp reduction in post-peak damage energy and an overall increase in damage severity. Under certain conditions, it is straightforward to produce a rapid release of energy, characterized as a rock burst phenomenon. This is a tentative mismatch with the idea of managing rock burst. It is suitable for rapid pressure relief and regional energy field regulation in small areas of the site.

### Adaptive integrated regulation system determination

**Figure 13:**
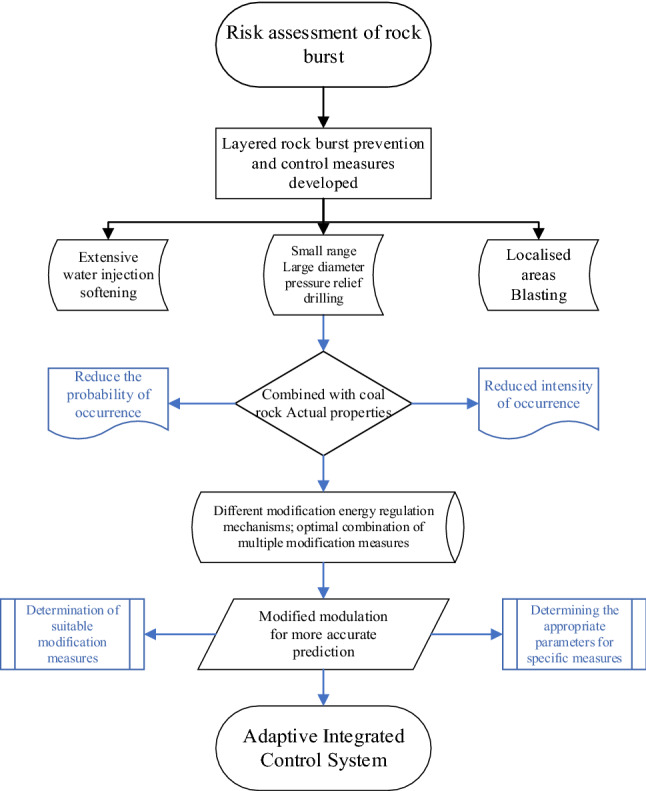
Framework diagram of adaptive comprehensive control system.

The complex mechanism of impact pressure initiation poses a severe test for the accurate prediction of the "time–space-intensity" of impact pressure occurrence. At the same time, numerous studies have shown that the rock damage system is a process of evolution from disorder to order, and the entropy of the whole process is constantly decreasing. Different modification measures, although increasing the complexity of the rock-damaged system, They can intervene in the system's evolution from disorder to order, the modification is for more accurate prediction. This requires a rational analysis of different modification and control measures based on the full use of the physical properties of the coal itself. By using of water injection softening standards in a large area, combined with a small size of large-diameter pressure relief borehole, the integrated formation of the adaptive control system is formed. And through the continuous optimization of measures to achieve the purpose of prediction and prevention of rock burst, effectively^26,27^. Framework diagram of the adaptive comprehensive control systems can be seen in Fig. 13.

## Analysis of the effect of filling high-speed energy-absorbing materials based on drilling modification and regulation

**Figure 14:**
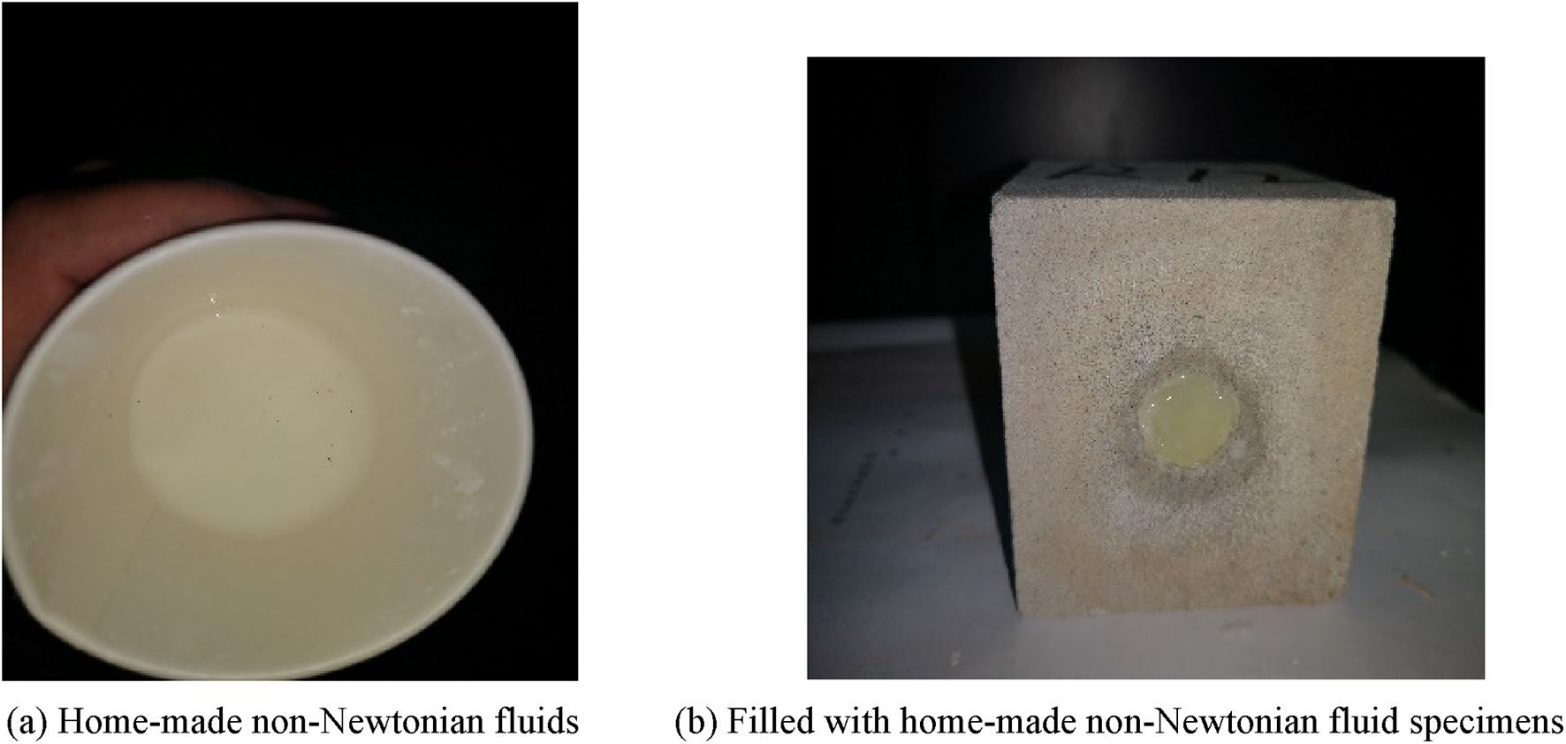
Preparation of Self-made Non-Newtonian Fluid Test and Sample Schematic.

**Figure 15:**
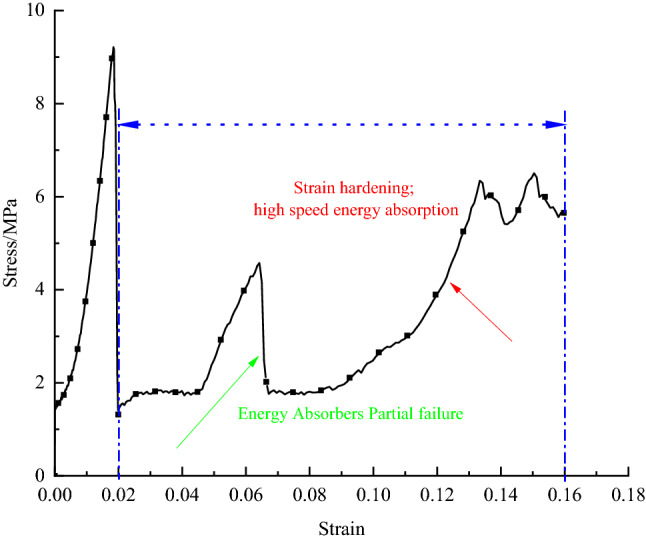
Load-bearing test curve of filled energy-absorbing materials under high-speed loading condition.

Based on the above analysis, the elastic energy stored before the peak of a purely porous rock sample is reduced. Still, the post-peak release energy is dramatic due to the artificially constructed soft surface. Different filling schemes and materials are shown in Table 6. To avoid the violent post-peak release energy of the whole rock sample, a filler material was considered to increase the post-peak disruption energy (non newtonian fluid energy-absorbing material, which is formed by mixing water and corn starch, as shown in Fig. 14), further reducing the violent post-peak release energy. As the rock burst is characterized by a rapid release of power, the non newtonian fluid is a good absorber of the high-velocity impact. Therefore, tests were designed to achieve the effect of non newtonian fluids by applying a high loading rate of 40 KN/s. The assessment of the energy absorption effect of bearing rock samples with borehole-filled non-Newtonian fluids was considered, ultimately providing new ideas for rock burst control. The test process curve is shown in Fig. 15.

**Table 6:** Sample number and filling materials in each state.

Specimen condition	The sample name	Specimen number	Filling properties
Nature intact	Rock sample	R_2-1, 2, 3_	Blank control I
Small aperture penetration		R_3-1, 2, 3_	Blank control II
Large aperture penetration		R_4-1, 2, 3_	Blank control III
small aperture non-penetration		R_5-1, 2, 3_	Blank control IV
Large aperture non-penetration		R_6-1, 2, 3_	Blank control V
Filled with non-Newtonian fluid energy absorbing material		R_7-1, 2, 3_	High speed energy absorption

In the uniaxial compression process, the rock sample under the borehole is not filled with material, because of its hole defects, resulting in the hole around the easy to produce a stress aggregation phenomenon, to produce crack initiation, expansion, and other chain damage reaction, and suddenly and promptly release the elastic strain energy stored before the original peak. In the process of deformation of the rock sample in the borehole filled with non newtonian fluids, although the stress concentration around the hole is effortless to induce crack initiation and expansion, the non newtonian fluids play a particular role in rigid support for the effect of high-speed loading. And it plays a role in raising the secondary energy storage threshold (peak elastic strain energy can be released) of the rock sample in the hole, which not only ensures that the elastic strain energy is not quickly removed, but also alleviates the sharpness of the release extent. Rock's samples filled with non-Newtonian fluid material can act as an energy absorber in the post-peak phase compared to intact rock samples, and those drilled with unfilled material. This is in line with the idea of preventing impact pressure.

## Conclusion

1. The physical and mechanical parameters of the rock samples were significantly altered under different conventional modification and conditioning measures. Compared with the control group, the average peak strength of the water-injection softened control rock samples decreased by 36.7% and 50.5%, the average peak strain decreased by 17.0% and 17.3%, and the average elastic modulus decreased by 32.0% and 49.7%, respectively. Compared with the control group, the average peak strength of the drill hole unloading control rock samples decreased by 11.1% and 41.2%, respectively; the average peak strain increased by 22.1% and decreased by 7.4%, and the average elastic modulus decreased by 64.6% and 47.5%, respectively. The average peak strength was reduced by 11.1% and 41.2%. The moderate peak strain was increased by 22.1% and decreased by 7.4%, and the average modulus of elasticity was decreased by 64.6% and 47.5%, respectively, compared with the control group.
2. The stress–strain curves of the rock samples under different modifications and control measures also go through the initial compression-density stage, elastic deformation stage, plastic deformation stage, yield damaged stage, and late damage stage. But the proportion of each step varies under different modifications and control measures, with emphasis on the yield damage stage and late damage stage. In addition, the energy evolution of the whole process also shows differences, especially in the yielding damage stage and the late damage stage. In contrast, the yielding damage stage provides favorable conditions for the accurate prediction of rock burst and the construction of an adaptive integrated control system.
3. Through a comprehensive analysis of the conventional modified energy regulation mechanism and the advantages, disadvantages and applicability of each regulation measure, a theoretical basis is provided for the rational determination of the adaptive integrated regulation system. Finally, by introducing non newtonian fluid energy-absorbing materials to fill the large-diameter pressure-relief borehole, the elastic energy released from instability is effectively absorbed. The feasibility of this approach is verified by combining the results of the indoor test data, which provides new ideas for further supplementary modified regulation schemes.

## References

[1] Wen J (2019). Rock burst risk evaluation based on equivalent surrounding rock strength. Int. J. Min. Sci. Technol..

[2] Wu Y (2019). Experimental study on the performance of rock bolts in coal burst-prone mines. Rock Mech. Rock Eng..

[3] Guo W (2017). Progressive mitigation method of rock bursts under complicated geological conditions. Int. J. Rock Mech. Min. Sci..

[4] Cao A (2016). Microseismic precursory characteristics of rock burst hazard in mining areas near a large residual coal pillar: A case study from Xuzhuang Coal Mine, Xuzhou, China. Rock Mech. Rock Eng..

[5] Di Y, Wang E (2021). Rock burst precursor electromagnetic radiation signal recognition method and early warning application based on recurrent neural networks. Rock Mech. Rock Eng..

[6] He J (2017). Rock burst assessment and prediction by dynamic and static stress analysis based on micro-seismic monitoring. Int. J. Rock Mech. Min. Sci..

[7] He S (2020). Coupled mechanism of compression and prying-induced rock burst in steeply inclined coal seams and principles for its prevention. Tunn. Undergr. Space Technol..

[8] Wu S, Wu Z, Zhang C (2019). Rock burst prediction probability model based on case analysis. Tunn. Underg. Space Technol..

[9] Khademian Z, Ugur O (2018). Computational framework for simulating rock burst in shear and compression. Int. J. Rock Mech. Min. Sci..

[10] Hirata A, Kameoka Y, Hirano T (2007). Safety management based on detection of possible rock bursts by AE monitoring during tunnel excavation. Rock Mech. Rock Eng..

[11] Chen B (2015). Rock burst intensity classification based on the radiated energy with damage intensity at Jinping II Hydropower Station, China. Rock Mech. Rock Eng..

[12] Zhao T (2018). Case studies of rock bursts under complicated geological conditions during multi-seam mining at a depth of 800 m. Rock Mech. Rock Eng..

[13] Wang G (2016). Evolution of stress concentration and energy release before rock bursts: Two case studies from Xingan Coal mine, Hegang, China. Rock Mech. Rock Eng..

[14] Li X (2016). Rock burst monitoring by integrated microseismic and electromagnetic radiation methods. Rock Mech. Rock Eng..

[15] Wang J (2016). Mechanism of rock burst occurrence in specially thick coal seam with rock parting. Rock Mech. Rock Eng..

[16] Akdag S (2018). Effects of thermal damage on strain burst mechanism for brittle rocks under true-triaxial loading conditions. Rock Mech. Rock Eng..

[17] Cai W (2018). A fuzzy comprehensive evaluation methodology for rock burst forecasting using microseismic monitoring. Tunn. Undergr. Space Technol..

[18] Rehbock-Sander M, Jesel T (2018). Fault induced rock bursts and micro-tremors - Experiences from the gotthard base tunnel. Tunn. Undergr. Space Technol..

[19] Cai M (2013). Principles of rock support in burst-prone ground. Tunn. Undergr. Space Technol..

[20] He S (2019). Precursor of spatio-temporal evolution law of MS and AE activities for rock burst warning in steeply inclined and extremely thick coal seams under caving mining conditions. Rock Mech. Rock Eng..

[21] Gong F (2019). A peak-strength strain energy storage index for rock burst proneness of rock materials. Int. J. Rock Mech. Min. Sci..

[22] Naji AM (2019). Geological and geomechanical heterogeneity in deep hydropower tunnels: A rock burst failure case study. Tunn. Undergr. Space Technol..

[23] Miao S (2016). Rock burst prediction based on in-situ stress and energy accumulation theory. Int. J. Rock Mech. Min. Sci..

[24] Cai W (2020). A monitoring investigation into rock burst mechanism based on the coupled theory of static and dynamic stresses. Rock Mech. Rock Eng..

[25] Qiu P (2021). Mitigating rock burst hazard in deep coal mines insight from dredging concentrated stress: A case study. Tunn. Undergr. Space Technol..

[26] Afraei S, Shahriar K, Madani SH (2018). Statistical assessment of rock burst potential and contributions of considered predictor variables in the task. Tunn. Undergr. Space Technol..

[27] Xiao YX (2016). Rock mass failure mechanisms during the evolution process of rockbursts in tunnels. Int. J. Rock Mech. Min. Sci..

